# Prediction of arterial extravasation in pelvic fracture patients with stable hemodynamics using coagulation biomarkers

**DOI:** 10.1186/s13017-019-0234-5

**Published:** 2019-03-19

**Authors:** Makoto Aoki, Takayuki Ogura, Shuichi Hagiwara, Mitsunobu Nakamura, Kiyohiro Oshima

**Affiliations:** 1 Department of Emergency Medicine, Gunma University Graduate School of Medicine, Gunma, Japan; 2 Advanced Medical Emergency Department and Critical Care Center, Japan Red Cross Maebashi Hospital, Gunma, Japan

**Keywords:** Trauma, Pelvic fracture, Coagulopathy, Arterial extravasation

## Abstract

**Background:**

Determining the presence of an active arterial hemorrhage in the acute phase is important as a treatment strategy in patients with pelvic fracture. The purpose of this study was to evaluate whether coagulation biomarkers could predict arterial extravasation, especially in pelvic fracture patients with stable hemodynamics.

**Methods:**

We studied patients with a pelvic fracture who had a systolic blood pressure above 90 mmHg and lactate level less than 5.0 mmol/L on hospital arrival. Patients were divided into two groups: those with arterial extravasation on enhanced computed tomography (CT) or angiography (extravasation [+] group) and those without arterial extravasation (extravasation [−] group). Coagulation biomarkers measured on arrival were statistically compared between the two groups. Predictive ability of arterial extravasation using coagulation biomarkers was evaluated by receiver-operating characteristic analyses provided area under the receiver-operating characteristic curves (AUROC) and diagnostic indicators with optimal cutoff point including sensitivity, specificity, positive and negative predictive values, and diagnostic odds ratio (DOR).

**Results:**

Sixty patients were analyzed. Fibrin degradation products (FDP), D-dimer, prothrombin time–international normalized ratio (PT–INR), and the ratio of FDP to fibrinogen were significantly higher in the extravasation (+) group than in the extravasation (−) group (FDP, 242 μg/mL [145–355] vs. 96 μg/mL [58–153]; D-dimer, 81 μg/mL [41–140] vs. 39 μg/mL [21–75]; PT–INR, 1.09 [1.05–1.24] vs. 1.02 [0.98–1.08]; and ratio of FDP to fibrinogen, 1.06 [0.85–2.01] vs. 0.46 [0.25–0.74]). The highest AUROC was with a ratio of FDP to fibrinogen of 0.777 (95% confidence interval, 0.656–0.898), and the highest predictive ability in terms of DOR was with a ratio of FDP to fibrinogen (sensitivity, 0.76; specificity, 0.76; DOR 9.90).

**Conclusion:**

Coagulation biomarker could predict of arterial extravasation in pelvic fracture patients with stable hemodynamics.

**Electronic supplementary material:**

The online version of this article (10.1186/s13017-019-0234-5) contains supplementary material, which is available to authorized users.

## Background

Pelvic fracture has been shown to be an independent risk factor for death after blunt trauma [1, 2]. It is associated with increased mortality in the blunt trauma population. In the acute phase of a pelvic fracture, arterial hemorrhage occurs from 3 to 20% of patients and induces hemodynamic instability [3–5]. The standard procedure for the detection of arterial hemorrhage has been contrast-enhanced computed tomography (CT). Arterial extravasation on contrast-enhanced CT is the most important predictor of the need for hemostatic treatment, such as transcatheter arterial embolization (TAE) [3]. However, contrast-enhanced CT has such problems as patients developing allergies to the contrast material [6], low sensitivity [7], and difficulties in radiographical interpretation [8, 9]. Low sensitivity may lead to a high false-negative rate for arterial extravasation, which may result in a misdiagnosis and delayed mortality [8]. The exact evaluation of arterial extravasation has differed between readers, which made the interpretation of contrast-enhanced CT more difficult.

In response, we report on the predictive nature of coagulation biomarkers for arterial extravasation in patients with pelvic fracture as a supplementary method of contrast-enhanced CT [10]. However, a practical problem in our previous study was that it included all pelvic fracture patients regardless of their hemodynamic status: In a real clinical situation, most clinicians will not hesitate to undertake hemostatic treatment of pelvic fracture cases with unstable hemodynamics. Recent trauma guidelines recommend that trauma patients with unstable hemodynamics undergo immediate hemostatic treatment and not contrast-enhanced CT after pelvis X-rays [11]. Therefore, determining whether using coagulation biomarkers can be a supplementary method of contrast-enhanced CT for pelvic fracture patients with stable hemodynamics is both practical and very important.

Thus, we investigated the usefulness of coagulation biomarkers to predict arterial extravasation in pelvic fracture patients with stable hemodynamics.

## Methods

### Study design

A double-center, retrospective, observational study was performed. The study protocol was approved by the institutional review board of Gunma University Hospital and Japan Red Cross Maebashi Hospital. The medical records of patients with a pelvic fracture, transferred to the emergency department (ED) of these hospitals between December 2009 and December 2016, were reviewed.

### Patient selection

Patients with a pelvic fracture who received prehospital treatment comprising only crystalloids and/or packed red blood cell infusions and who were tested for coagulation biomarkers on ED arrival were included. We defined exclusion criteria according to that of our previous study [10]: (1) abbreviated injury scale (AIS) scores in another region that was higher than the pelvis AIS score, (2) arterial extravasation in regions other than the pelvis, and (3) unknown time of trauma occurrence. In addition, we excluded pelvic fracture patients with unstable hemodynamics (systolic blood pressure below 90 mmHg on ED arrival and/or lactate level above 5.0 mmol/L).

### Analytic variables

The following parameters were obtained on ED arrival: patient demographics, use of anticoagulation, and/or antiplatelet drugs, trauma mechanism, vital signs at ED arrival (the Glasgow Coma Scale, systolic blood pressure, heart rate, and respiratory rate), results of blood tests (hemoglobin and lactate levels, levels of fibrin degradation products [FDP], D-dimer and fibrinogen, prothrombin time–international normalized ratio [PT–INR]), AIS for each body region, injury severity score (ISS), patterns of pelvic fracture classified by Young and Burgess classification [12], World Society of Emergency Surgery (WSES) classification [13], angiography for the pelvis, treatment for pelvic fracture (TAE, external fixation, preperitoneal packing, and amount of blood transfusion within 24 h (in Japan, 1 U of packed red blood cells is approximately 140 mL), time course (door to CT time and door to angiography time), and mortalities (24 h and 30 days). With regard to analytic variables, the ratio of FDP to fibrinogen (FDP/fibrinogen) was also calculated, as with our previous study [10]. FDP and D-dimer were measured using an immunoturbidimetric method and using Cs-2000i and Cs-5100 systems (Sysmex Corporation, Kobe, Japan). It took about 15 min to gain the results of coagulation biomarkers by the immunoturbidimetric method.

### Trauma workflow of pelvic fracture patient

During the study periods, strategies, diagnostic devices, and treatment options as to pelvic fracture were not changed. All patients who were suspected of having a pelvic fracture with stable hemodynamics underwent a contrast-enhanced CT in hospital. Contrast-enhanced CT was performed in arterial and portal venous phases. If arterial extravasation was detected on contrast-enhanced CT, we basically performed angiography. In addition, angiography was performed on patients who progressed to unstable hemodynamics and/or had a progressive retroperitoneal hematoma without obvious arterial extravasation on contrast-enhanced CT. If necessary in the acute phase after TAE, external pelvic fixation (damage control orthopedics) was performed. Basically, pelvic fracture patients were treated by transfusion and resuscitated by the use of resuscitation of endovascular occlusion of the aorta if necessary until TAE is completed.

### Definition of arterial extravasation

Arterial extravasation was defined as extravascular high attenuating regions with attenuation similar to or greater than that of the aorta on arterial phase images, and the increase of extravasation during the portal phase compared the arterial phase. Arterial extravasation on contrast-enhanced CT and angiography was analyzed by at least one radiologist.

### Definition of reference standard (extravasation [+] and extravasation [−])

We divided the studied patients into two groups: The extravasation (+) group was defined as having arterial extravasation on contrast-enhanced CT and/or angiography, and the extravasation (−) group was defined as not having arterial extravasation on contrast-enhanced CT and/or angiography. The emergency physicians and/or the interventional radiologist made the decision to proceed to angioembolization.

### Statistical analysis

Data are expressed as the median (interquartile reference; IQR). Comparisons of each parameter between extravasation (+) and extravasation (−) groups were performed using Mann–Whitney *U*, and chi-squared tests or Fisher’s exact test. The efficacy of predicting arterial extravasation was evaluated using the area under the receiver-operating characteristic curves (AUROC), with low, medium, and high accuracy defined as < 0.7, ≥ 0.7 to < 0.9, and ≥ 0.9, respectively [14]. The optimal cutoff point was defined by the maximum of the sum of sensitivity and specificity using the Youden index approach. An ordinal 2-by-2 cross-tabulation analysis for diagnostic purposes estimated sensitivity, specificity, positive and negative predictive value, positive and negative likelihood ratio, and diagnostic odds ratio (DOR). Statistical analysis was performed with IBM SPSS Statistics version 25.0 (Armonk, NY, USA). Statistical significance was defined as a two-sided *p* value < 0.05.

### Subgroup analysis

We performed a subgroup analysis. Subgroup analysis included pelvic fracture patients with stable hemodynamics that had AIS scores in another region that was higher than the pelvis AIS score.

## Results

**Fig. 1:**
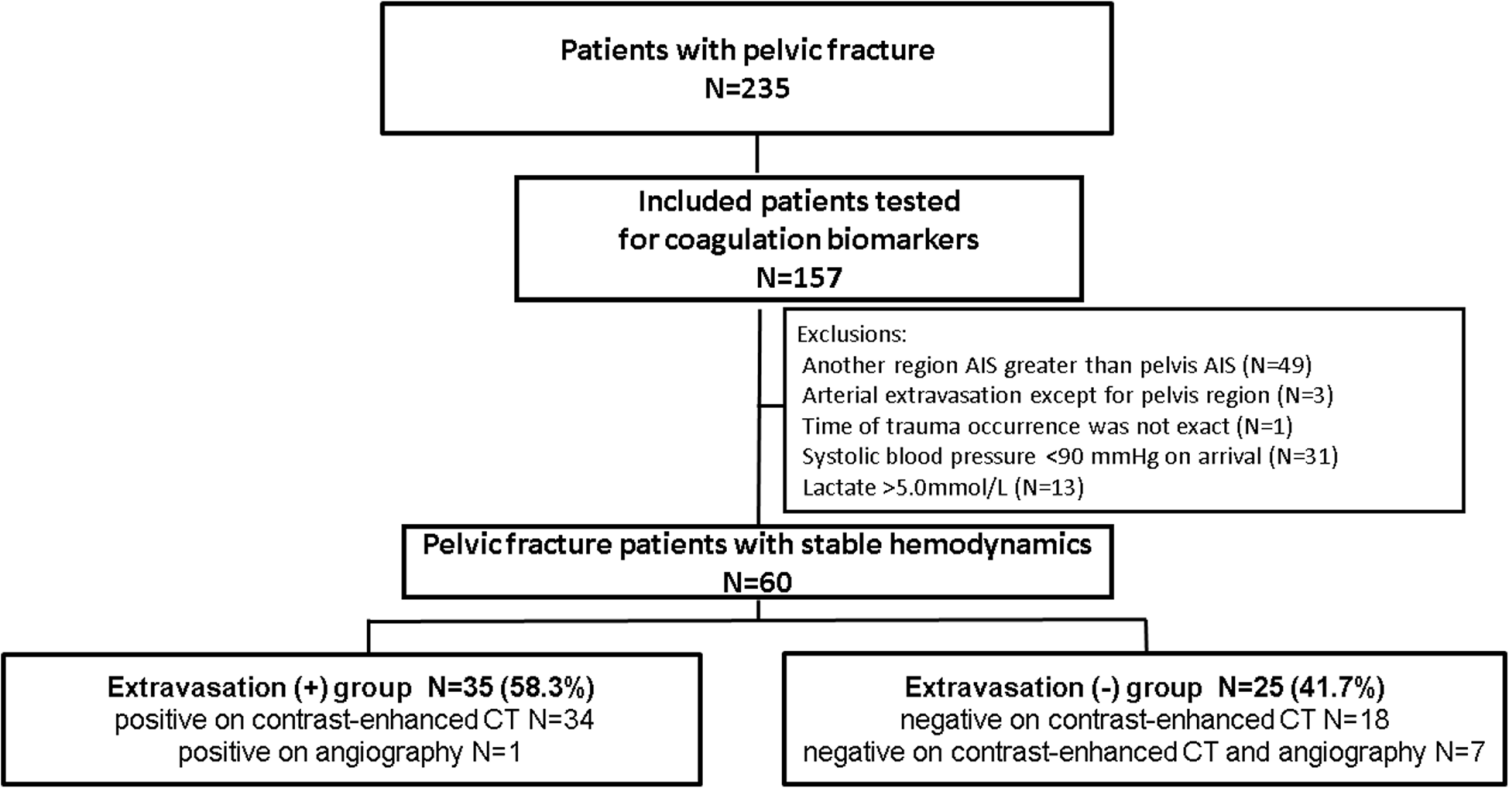
Patients flow chart showing their selection and groupings. Supplemental digital content 1: AUC and cutoff points of parameters to predict arterial extravasation in pelvic fracture patients included patients with a greater AIS region other than the pelvis

A patient flow chart is shown in Fig. 1. Between December 2009 and December 2016, 235 patients with pelvic fracture were transferred and admitted to hospitals. Of these 235 patients, 157 patients were tested for coagulation biomarkers. Forty-nine patients with AIS scores that were greater in another region than the pelvis, three patients with an arterial extravasation in a non-pelvic region, one patient with an unknown time of trauma occurrence, and 44 patients with unstable hemodynamics were excluded. Finally, 60 pelvic fracture patients with stable hemodynamics were studied. The extravasation (+) group included 35 patients: 34 with contrast extravasation on contrast-enhanced CT, and one with contrast extravasation only found on angiography. The extravasation (−) group included 25 patients: none had contrast extravasation on contrast-enhanced CT, and seven underwent angiography without evidence of contrast extravasation.

Patients’ baseline characteristics between extravasation (+) and the extravasation (−) groups are shown in Table 1. The median age was 67 (54–80) years. Median ages were 73 (61–82) years and 63 (42–70) years in the extravasation (+) group and in the extravasation (−) group, respectively). Thirty-eight patients (63.3%) were males. Five patients (14.3%) in extravasation (+) group had use of antiplatelet drugs (*p* = 0.043). The leading cause of the trauma mechanism was motor vehicle collision (65.0%), with falls (28.3%) following. Concerning the vital signs taken in each ED, only the Glasgow Coma Scale was significantly lower in the extravasation (+) group than in the extravasation (−) group (14 [13–15] vs. 15 [14, 15]; *p* = 0.033).

**Table 1:** Baseline characteristics of study patients

Variables	Extravasation (+) *n* = 35	Extravasation (−) *n* = 25	*p* value
Age, years^a^	73 (61–82)	63 (42–70)	0.097
Gender (male), *n* (%)^b^	18 (51.4)	20 (80.0)	0.031
Use of anticoagulation, antiplatelet drugs, *n* (%)	5 (14.3)	0 (0.0)	0.048
Trauma mechanism, *n* (%)^b^			0.105
Motor vehicle	27 (77.1)	12 (48.0)	
Fall	7 (20.0)	10 (40.0)	
Sport	0 (0.0)	1 (4.0)	
Others	1 (2.9)	2 (8.0)	
Vital signs at ED			
GCS^a^	14 (13–15)	15 (14–15)	0.033
sBP, mmHg^a^	124 (109–133)	130 (112–161)	0.205
HR, bpm^a^	81 (71–94)	76 (68–88)	0.304
RR, bpm^a^	21 (17–24)	18 (14–20)	0.270
Blood Test			
Hemoglobin level, g/dL^a^	11.7 (10.2–13.5)	12.9 (11.8–14.3)	0.078
Lactate level, mmol/L^a^	2.25 (1.58–2.86)	2.02 (1.40–2.70)	0.486
FDP, μg/mL^a^	242 (145–355)	96 (58–153)	< 0.001
D-dimer, μg/mL^a^	81 (41–140)	39 (21–75)	0.006
Fibrinogen, mg/dL^a^	225 (167–295)	229 (198–266)	0.931
PT–INR^a^	1.09 (1.05–1.24)	1.02 (0.98–1.08)	0.003
Ratio of FDP to fibrinogen^a^	1.06 (0.85–2.01)	0.46 (0.25–0.74)	< 0.001
AIS for each region			
Head (*n* = 26)^a^	3 (3–4)	3 (2–4)	0.024
Chest (*n* = 25)^a^	3 (3–4)	3 (3–4)	0.023
Abdomen (*n* = 12)^a^	3 (2–4)	3 (3–4)	0.540
Extremities and the pelvis (*n* = 60)^a^	4 (4–5)	3 (3–4)	< 0.001
ISS^a^	32 (25–44)	14 (9–27)	< 0.001
Patterns of pelvic fracture^b^			
Young and Burgess			0.021
LC1, *n* (%)	0 (0.0)	4 (16.0)	
LC2, *n* (%)	12 (34.3)	9 (36.0)	
LC3, *n* (%)	16 (45.7)	4 (16.0)	
APC1, *n* (%)	0 (0.0)	0 (0.0)	
APC2, *n* (%)	1 (2.9)	0 (0.0)	
APC3, *n* (%)	0 (0.0)	0 (0.0)	
VS, *n* (%)	6 (17.1)	8 (32.0)	
WSES classification			0.011
1, *n* (%)	0 (0.0)	4 (16.0)	
2, *n* (%)	29 (82.9)	13 (52.0)	
3, *n* (%)	6 (17.1)	8 (32.0)	
Diagnostic procedure			
Angiography for the pelvis, *n* (%)^b^	33 (94.3)	7 (28.0)	< 0.001
Treatment for pelvic fracture			
TAE, *n* (%)^b^	33 (94.3)	4 (16.0)	< 0.001
External fixation, *n* (%)^b^	2 (6.1)	1 (5.0)	1.000
Preperitoneal packing, *n* (%)^b^	0 (0.0)	0 (0.0)	N.A
Amount of blood transfusion within 24 h, units^a^	4 (0–14)	0 (0–0)	0.002
Time course			
Door to CT time, min	22 (17–32)	23 (19–32)	0.722
Door to angiography time, min	92 (81–116)	71 (53–92)	0.059
Mortality			
24-h mortality, *n* (%)^b^	0 (0.0)	0 (0.0)	1.000
30-day mortality, *n* (%)^b^	4 (11.4)	0 (0.0)	0.216

^a^Mann–Whitney *U* test

^b^χ^2^ test or Fisher’s exact test

*ED* emergency department, *GCS* Glasgow Coma Scale, *sBP* systolic blood pressure, *HR* heart rate, *RR* respiratory rate, *FDP* fibrin degradation products, *PT–INR* prothrombin time–international normalized ratio, *WSES* World Society of Emergency Surgery, *AIS* abbreviated injury scale, *TAE* transcatheter arterial embolization, *ISS* injury severity score, *N.A* not applicable

**Fig. 2:**
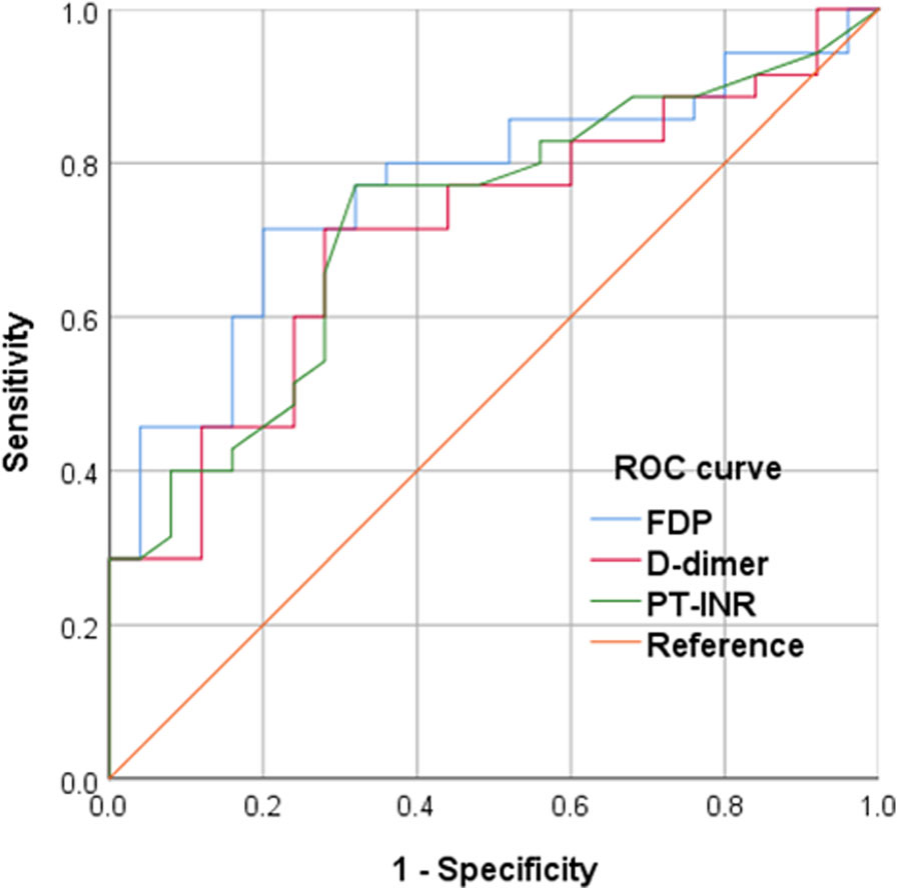
Univariate receiver-operating characteristic (ROC) curve analysis for arterial extravasation. FDP fibrin degradation products, PT–INR prothrombin time–international normalized ratio, ROC receiver-operating characteristic

Concerning blood tests, there was no significant difference in the hemoglobin level between the extravasation (+) group and extravasation (−) group (11.7 [10.2–13.5] g/dL vs. 12.9 [11.8–14.3] g/dL; *p* = 0.078). The FDP (242 [145–355] μg/mL vs. 96 [58–153] μg/mL; *p* < 0.001), D-dimer (81 [41–140] μg/mL vs. 39 [21–75] μg/mL; *p* = 0.006), PT–INR (1.09 [1.05–1.24] vs. 1.02 [0.98–1.08]; *p* = 0.003) levels and ratio of FDP to fibrinogen (1.06 [0.85–2.01] vs. 0.46 [0.25–0.74]; *p* < 0.001) were significantly higher in the extravasation (+) group. The AUROC for FDP, D-dimer, PT–INR and the ratio of FDP to fibrinogen are shown in Table 2. The ratio of FDP to fibrinogen showed the highest AUROC (0.777 [0.656–0.898]), and the highest diagnostic ability in terms of DOR (sensitivity 0.76, specificity 0.76, and DOR 9.90. In a subgroup analysis, FDP and D-dimer had the highest DOR (please see Additional file 1: Table S1). The ROC curves for FDP, D-dimer, and PT–INR are shown in Fig. 2.

**Table 2:** AUROC of parameters to predict arterial extravasation in pelvic fracture patients

Variables	FDP	D-dimer	PT–INR	Ratio of FDP to fibrinogen
AUC (95% CI)	0.767 (0.646–0.887)	0.710 (0.579–0.841)	0.725 (0.596–0.853)	0.777 (0.656–0.898)
Cutoff point	179.2 μg/mL	52.0 μg/mL	1.05	0.79
Sensitivity (%)	71	71	77	76
Specificity (%)	80	72	68	76
Positive predictive value (%)	83	78	77	77
Negative predictive value (%)	67	64	68	68
Positive likelihood ratio	3.55	2.54	2.40	3.17
Negative likelihood ratio	0.36	0.40	0.34	0.32
DOR	9.86	6.35	7.06	9.90

*AUROC* area under the receiver-operating characteristic curves, *CI* confidence interval, *FDP* fibrin degradation products, *PT–INR* prothrombin time–international normalized ratio, *DOR* diagnostic odds ratio

The extravasation (+) group had a significantly higher AIS score for the head (3 [3, 4] vs. 3 [2–4]; *p* = 0.024), chest (3 [3, 4] vs. 3 [3, 4]; *p* = 0.023), and the extremities and pelvis (4 [4, 5] vs. 3 [3, 4]; *p* < 0.001; Table 1). The extravasation (+) group had a significantly higher ISS (32 [25–44] vs. 14 [9–27]; *p* < 0.001; Table 1). There was a significant difference in pelvic fracture pattern in Young and Burgess classification (*p* = 0.021) and WSES classification (*p* = 0.011). As for treatment, 94% (33/35) of the extravasation (+) group had angiography and all of the patients who had angiography underwent angiography with TAE, and 60% (21/35) of the extravasation (+) group had a blood transfusion within 24 h of the time of the injury. As for time course, the median door to CT time was 22 [18–32] minutes and median door to angiography time was 90 [72–110] minutes. All 25 extravasation (−) patients survived. Among the 35 extravasation (+) patients, all patients survived within the first 24 h; however, four patients died within 30 days from trauma occurrence for non-hemorrhagic reasons.

## Discussion

### Brief summary

This study demonstrated the association of arterial extravasation in pelvic fracture patients with stable hemodynamics and coagulation biomarkers. Coagulation biomarkers could predict of arterial extravasation in pelvic fracture patients with stable hemodynamics.

### Characteristics of coagulation biomarkers that predict arterial extravasation

Since the usefulness of TAE for pelvic fracture was first reported [13–15], investigators have debated which patients with pelvic fracture should undergo this procedure [15–18]. Pelvic fracture patients with unstable hemodynamics were thought to require a prompt hemostatic procedure. However, it is clinically important to predict arterial bleeding in pelvic fracture patients with stable hemodynamics and to judge the necessity of hemostatic treatment before the collapse of their hemodynamics. Arterial extravasation is an important indication for needing TAE [19]. The most important finding of this study was that arterial extravasation in pelvic fracture patients with stable hemodynamics could be predicted by coagulation biomarkers. We could predict of arterial extravasation by coagulation biomarkers before CT scanning, and this perception alerted the later collapse of hemodynamics. Actually, the 15 patients of extra (+) became unstable hemodynamics before completion of hemostatic treatment. The diagnostic abilities of coagulation biomarkers were medium accuracy [14] and not so high (Table 2 and Fig. 2); therefore, a combination of contrast-enhanced CT and coagulation biomarkers was thought to be realistic and practical. For instance, we could judge whether the pelvic fracture patient with stable hemodynamics should undergo enhanced CT or not by coagulation biomarker when we doubted that the patient has arterial extravasation from initial physical examination and/or initial pelvis X-ray.

We performed a subgroup analysis to maintain the clinical generality of the study results. For the subgroup analysis, we evaluated whether coagulation biomarkers could be applied to patients with a pelvic fracture who had a more severe injury of another body part. Coagulation biomarkers were obviously affected by the presence of another anatomical injury, except for the pelvis. As a result, the predictive nature of coagulation biomarkers for arterial extravasation in pelvic fracture was decreased; however, this was of a medium level of accuracy [10]. In a clinical situation, patients with severe pelvic fracture often have another more severely injured body region. Subgroup analysis revealed that coagulation biomarkers may be useful in predicting arterial extravasation among multiple trauma pelvic fracture patients.

### Possible explanations and implications: the necessity of hemostatic intervention for pelvic fracture patients with stable hemodynamics

Whether arterial bleeding in pelvic fracture patients with stable hemodynamics needs TAE is contentious. It was previously reported that only 23% of arterial extravasation cases observed on contrast-enhanced CT needed TAE [20], and we agree that all arterial extravasation cases do not need TAE. The strength of this study was that we could repeatedly predict arterial bleeding in pelvic fracture patients with stable hemodynamics and the necessity of TAE based on coagulopathy. Since the concept of trauma-induced coagulopathy has been advocated, coagulopathy accompanying trauma is associated with mortality [21–23]. Hemorrhage and coagulopathy affect each other. Therefore, hemorrhage induces more severe coagulopathy and coagulopathy induces more active hemorrhage in the absence of prompt treatment, to the point where a patient’s hemodynamics will eventually collapse. In this study, the extravasation (+) group showed arterial hemorrhage and coagulopathy; both of these are thought to deteriorate together. Of the extravasation (+) group with enhanced CT, 94% of patients (32/34) needed TAE, which indicated clinicians judged the arterial extravasation with enhanced CT could not conservatively be arrested. The high positive rate of arterial extravasation with angiography among arterial extravasation with enhanced CT may be because of coagulopathy [24]. Coagulopathy was more deteriorated in five patients of the extravasation (+) due to anticoagulation and/or antiplatelet drugs (Table 1). Finally, 15 patients of the extravasation (+) group became unstable hemodynamics; however, there was no hemorrhage-related 24-h mortality.

This study has several limitations. First, the values measured for coagulation biomarkers could have been affected by the timing of the test. In this study, we excluded a patient for whom the time of trauma was unknown; however, we did not take into account the time from trauma occurrence to testing. Second, we did not account for the amount of prehospital intravenous fluids given to patients. The administration of fluids can dilute the blood and affect the value of coagulation biomarkers. Third, we had no information about using tranexamic acid in this retrospective study. Tranexamic acid was thought to be affecting the coagulopathic state; therefore, this was an important limitation [25]. Fourth, the number of patients in this study was not large and the validation of study’s result would be necessary.

## Conclusion

Coagulation biomarkers could predict arterial extravasation of pelvic fracture patients with stable hemodynamics. Early recognition of coagulopathy could urge us to undertake a prompt enhanced CT and/or TAE before the hemodynamics of patients with a pelvic fracture become unstable.

## Additional file

Additional file 1: **Table S1.** AUROC of parameters to predict arterial extravasation in pelvic fracture patients including patients with a greater AIS region other than the pelvis (*n* = 92). (DOCX 14 kb)

## Abbreviations

AIS: Abbreviated injury scale
AUROC: Area under the receiver-operating characteristic curves
CT: Computed tomography
DOR: Diagnostic odds ratio
ED: Emergency department
FDP: Fibrin degradation products
IQR: Interquartile reference
ISS: Injury severity score
PT-INR: Prothrombin time–international normalized ratio
TAE: Transcatheter arterial embolization
