# Supplementary material for: Prediction of arterial extravasation in pelvic fracture patients with stable hemodynamics using coagulation biomarkers
Source: World J Emerg Surg. 2019 Mar 19;14:14. doi: 10.1186/s13017-019-0234-5 (PMC6425576; doi:10.1186/s13017-019-0234-5)
Supplement: Supplementary file 1 — Table S1. AUROC of parameters to predict arterial extravasation in pelvic fracture patients including patients with a greater AIS region other than the pelvis (n = 92). (DOCX 14 kb) [file 13017_2019_234_MOESM1_ESM.docx]

Additional file1: Table S1 AUROC of parameters to predict arterial extravasation in pelvic fracture patients including patients with a greater AIS region other than the pelvis (n=92)

| Variables | FDP | D-dimer | PT–INR | Ratio of FDP  to fibrinogen |
| --- | --- | --- | --- | --- |
| AUC (95% CI) | 0.724 (0.618–0.829) | 0.710 (0.602–0.818) | 0.667 (0.556–0.778) | 0.711 (0.601–0.820) |
| Cut-off point | 207.9μg/mL | 71.5μg/mL | 1.05 | 0.81 |
| Sensitivity (%) | 73 | 73 | 84 | 84 |
| Specificity (%) | 71 | 71 | 50 | 50 |
| Positive predictive value | 70 | 70 | 61 | 61 |
| Negative predictive value | 74 | 74 | 77 | 77 |
| Positive likelihood ratio | 2.51 | 2.51 | 1.68 | 1.68 |
| Negative likelihood ratio | 0.38 | 0.38 | 0.32 | 0.32 |
| DOR | 6.61 | 6.61 | 5.25 | 5.25 |

AUROC, area under the receiver-operating characteristic curves; CI, confidence interval; FDP, fibrin degradation products; PT–INR; prothrombin time–international normalized ratio; DOR, diagnostic odds ratio
